# Long-period grating optical fiber sensor for IgG-type monoclonal protein measurement

**DOI:** 10.3389/fbioe.2026.1837135

**Published:** 2026-07-15

**Authors:** Peizhou Wu, Stephen P. Morgan, Ricardo Correia, Serhiy Korposh

**Affiliations:** Faculty of Engineering, Optics and Photonics Research Group, University of Nottingham, Nottingham, United Kingdom

**Keywords:** biosensor, light chains, long-period grating, monoclonal proteins, multiple myeloma

## Abstract

Measuring monoclonal proteins (M-proteins) plays an important role in the diagnosis and monitoring of plasma cell dyscrasias. Measurements of M-proteins are mostly conducted by electrophoresis, which requires complex sample processing and takes several hours to obtain a result. In this work, IgG kappa M-protein (IgG_κ_) and IgG lambda M-protein (IgG_λ_) sensors are developed based on a long-period grating optical fiber sensor. The IgG_κ_ sensor has a limit of detection (LoD) of 2.78 μg/mL and a selectivity of 9.73. The IgG_λ_ sensor has an LoD of 2.75 μg/mL and a selectivity of 27.9. The sensor has sufficient sensitivity to detect clinically relevant M-protein levels, highlighting its significant potential as a rapid, easy-to-use point-of-care testing (POCT) platform with strong clinical applicability in healthcare.

## 1 Introduction

Plasma cell dyscrasias can cause the malignant proliferation of B cells (Kyle and Greipp, 1988; Rajkumar, 2024). B cells produce immunoglobulins (Ig) (van de Donk et al., 2021; Lydyard et al., 2011), which are constituted of heavy chains and light chains (van de Donk et al., 2021; Lydyard et al., 2011). There are five types of heavy chains (α, γ, ε, δ, and µ chains) and two types of light chains (κ and λ chains) (Lydyard et al., 2011). A heavy chain can bind to only one type of light chain to form a whole Ig. For example, a γ chain can only bind to λ chains to form IgG [human IgG lambda (IgG_λ_)]; another γ chain can only bind to a κ chain to form IgG [human IgG kappa (IgG_κ_)]. For healthy people, the seven types of chains (five heavy chains and two light chains) are balanced. However, in people with plasma cell dyscrasias, one type of chain exceeds the normal range, while the other types of chains remain within the normal range or decrease (Soma, 2022). As a result, the corresponding immunoglobulins exceed the normal range (up to 30 mg/ml). For example, in patients with IgG_κ_ myeloma, excessive γ chains are produced, and these γ chains bind only to the κ chain. As a result, IgG_κ_ exceeds the normal level.

The proteins produced by malignant B cells are also called monoclonal proteins (M-proteins), which include full immunoglobulins and their fragments. IgG_λ_ and IgGκ are two types of M-proteins. In clinical practice, the level of M-proteins is used as a diagnostic criterion for monoclonal gammopathy of undetermined significance (MGUS) and smoldering multiple myeloma (SMM) (Rajkumar and Kumar, 2016; Morrison et al., 2019). MGUS and SMM are considered early stages of multiple myeloma (MM). Measuring the concentration of M-protein in a patient’s serum is the gold standard for monitoring disease progression (Udd et al., 2017). MGUS, SMM, and MM are easily neglected during medical checkups as they are rare diseases and their symptoms are nonspecific, including bone pain, fatigue, and weight loss, which can be easily attributed to concurrent health problems. A rapid, easy-to-use M-protein sensor could facilitate diagnosis.

M-proteins are detected by immunofixation (O’connell et al., 2005; Lakshminarayanan et al., 2007). Current methods require complex sample processing and bulky equipment, taking several hours to produce results. Therefore, there is a critical need to transition these diagnostics toward point-of-care testing (POCT). Optical fiber sensors possess advantages such as light weight, small size, and immunity to electromagnetic interference (Correia et al., 2018), making them ideal candidates for rapid testing and ease of use in clinical settings. To directly address the clinical demand for advanced diagnostics, Table 1 quantitatively benchmarks the performance of the proposed optical fiber sensor against established and emerging central laboratory techniques in terms of sensitivity, specificity, time, and cost.

**TABLE 1:** Comparison of M-protein detection technologies.

Technology	Target specificity	Sensitivity (LoD)	Time to result	Equipment and POCT capability	Estimated cost/ translation barrier
Serum protein electrophoresis (SPEP) (O’connell et al., 2005)	Low (detects bulk M-protein; cannot type clones)	Low (∼1,000 μg/mL)	Hours	Bulky/ central lab (no POCT)	Low per test (∼£25 – £75)
Immunofixation (IFE) (Lakshminarayanan et al., 2007; Csako, 2012)	High (standard clinical typing of heavy/light chains)	Moderate (∼100 μg/mL–250 μg/mL)	Hours	Bulky/ central lab (no POCT)	Moderate per test (∼£80 – £200)
Mass spectrometry (Zajec et al., 2020)	Very high (exact molecular mass differentiation)	Very high (∼1 μg/mL–10 μg/mL)	Hours to days	Highly complex (no POCT)	Very high capital (exceeds £120k+)
U-shaped SPR biosensor (Du and Wang, 2025)	Low (total IgG only; lacks light-chain specificity)	Extreme (∼1.28 ng/mL)	Minutes	Benchtop setup (potential POCT)	Moderate (requires complex nanoparticle coatings)
DTP-LPFG biosensor (Esposito et al., 2021)	Low (total IgG only; lacks light-chain specificity)	Extreme (∼0.16 ng/mL)	Minutes	Benchtop setup (potential POCT)	Moderate (requires extreme tuning precision)
Proposed LPG sensor [this work]	High (explicitly types IgGκ and IgGλ clones)	High (balanced) (∼2.75–2.78 μg/mL)	∼25 minutes	Compact/ portable spectrometer (POCT ready)	Very low (<£4 per disposable optical fiber)

**FIGURE 1:**
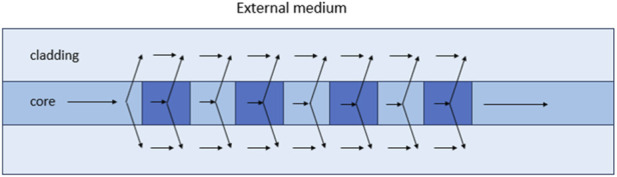
Schematic illustration of a long-period grating demonstrating the coupling of the core mode to the set of cladding modes.

A long-period grating (LPG) optical fiber is a configuration of an optical fiber sensor that has great potential as a biosensor. An LPG is fabricated in a single-mode optical fiber by modulating the refractive index (RI) of the core. When light propagates along the fiber, the LPG causes the core mode to couple into cladding modes (Figure 1). Therefore, attenuation bands are formed in the transmission spectrum. The wavelengths of attenuation bands are determined using Equation 1 (James and Tatam, 2003):

$$\lambda_{x}=\left({n_{eff}}^{co}-{n_{eff}}^{cl,x}\right)\Lambda .$$ (1)

Here, ${n_{eff}}^{co}$ is the effective RI of the core mode, ${n_{eff}}^{cl,x}$ is the effective RI of the *x*th cladding mode, and Λ is the grating period. Biosensors for measuring human antibodies have been designed based on LPGs (Liu et al., 2018; Wu et al., 2021). Bio-receptors that binding to the heavy chains of antibodies are immobilized on the surface of LPGs. When the target antibodies attach to the bio-receptors, the RI on the surface increases, causing shifts in the wavelength of the attenuation bands.

Quantitatively, it is well-known (James and Tatam, 2003; Wu et al., 2021) that the mechanism of this wavelength shift is governed by the interaction between the evanescent field of the cladding modes and the surrounding medium. When the target M-proteins bind to the immobilized bio-receptors, there is a physical accumulation of protein mass on the fiber surface. Because protein molecules possess a significantly higher refractive index than the surrounding aqueous buffer, this surface accumulation increases the local external refractive index (n_*ext*_) within the penetration depth of the evanescent field. This localized increase in n_*ext*_ directly modifies the effective refractive index of the affected cladding modes (n_eff_^cl,x^), while the core mode (n_eff_^co^) remains largely shielded and unaffected. According to the phase-matching condition expressed in Equation 1, any perturbation in n_eff_^cl,x^ alters the effective index difference between the core and cladding modes, thereby inducing a proportional, measurable shift in the resonant wavelength (l). Consequently, the sensor quantitatively translates the physical nanoscale buildup of antigen–antibody complexes into distinct optical spectral shifts.

Although optical fiber biosensors have been widely utilized for the label-free detection of generic proteins and whole antibodies, their application in the diagnosis of plasma cell dyscrasia has remained fundamentally limited because of the lack of clonality-specific targeting. Conventional optical immunosensors primarily utilize bio-receptors directed against heavy chains (e.g., anti-IgG) to quantify the total circulating antibody concentrations. However, diagnosing multiple myeloma and establishing the critical IgG_κ_/IgG_λ_ ratio requires the explicit differentiation of light chains. The fundamental innovation of this work lies in engineering an LPG platform that transitions from generic heavy-chain detection to highly specific light-chain targeting. By uniquely functionalizing the LPG surface with anti-IgG_κ_ and anti-IgG_λ_ receptors, this platform represents, to the best of our knowledge, the first optical fiber biosensor that is explicitly capable of directly subtyping M-proteins. This target specificity uniquely bridges the gap between highly sensitive photonic technologies and the precise clinical requirements of point-of-care monoclonal gammopathy typing, thus offering a rapid, automated alternative to the labor-intensive immunofixation electrophoresis (IFE) currently relied upon in central laboratories.

While the current study establishes the foundational proof of concept for M-protein detection in controlled buffer systems, the practical applicability of this technology in clinical settings requires robust performance in complex biological matrices. Previous studies from our group have extensively demonstrated the robustness of functionalized LPG optical fiber platforms in complex media, specifically proving their high selectivity for whole immunoglobulins, such as IgM, IgA, and IgG, against complex biological backgrounds (Wu et al., 2021). The present study directly expands upon that established foundation. Having previously proven the platform’s resilience to matrix effects and cross-interference from other immunoglobulin classes, we now demonstrate the capability to selectively measure and differentiate the specific light chains (IgG_κ_ and IgG_λ_) required for clinical M-protein typing. Building upon this established optical architecture (Korposh et al., 2014), future work will focus on validating these specific light-chain sensors in patient-derived serum samples to fully benchmark their clinical utility alongside traditional immunofixation.

## 2 Methodology

### 2.1 Materials

Anti-human λ chain antibody (bound and free) produced in goat (anti-λ), anti-human κ chain antibody (bound and free) produced in goat (anti-κ), anti-human IgG antibody (Fc-specific) produced in goat (anti-IgG), borate buffer saline, albumin from human serum (HSA), (3-aminopropyl)triethoxysilane (APTES), glutaraldehyde solution (50 wt% in H_2_O), sulfuric acid (H_2_SO_4_, 95%–98%), and hydrogen peroxide solution (H_2_O_2_, 35%) were purchased from Merck, United Kingdom. The anti-λ and anti-κ are bio-receptors for the IgG_λ_ and IgG_κ_ chains, respectively. IgG_κ_ and IgG_λ_ were purchased from Cambridge Bioscience, United Kingdom.

### 2.2 Experimental set-up and protocols

**FIGURE 2:**
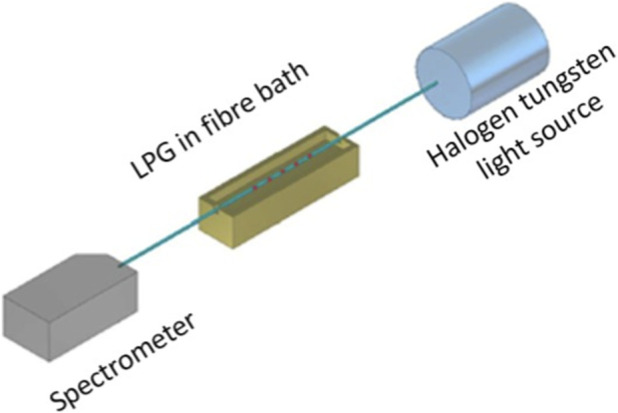
LPG placed in a homemade Teflon fiber bath holder [49 mm (L) × 3 mm (W) × 2 mm (D)].

An LPG with a length of 3 cm is fabricated in PS750 photosensitive fiber using a 266 nm Nd:YAG laser (Q-Smart 450, Quantel). For testing the response, the LPG is placed in a custom-made Teflon fiber bath holder [49 mm (L) × 3 mm (W) × 2 mm (D), Figure 2].

One end of the LPG is connected to a halogen–tungsten light source (Ocean Optics, HL2000), while the other is connected to a spectrometer (Ocean Optics, HR4000). When fabricating M-protein sensors, the following protocol is used:

1. An LPG is immersed in piranha solution for 20 min to remove organic contaminants and generate hydroxyl groups on the surface. Then, it is washed with deionized (DI) water and dried with nitrogen.
2. The LPG is immersed in an APTES solution (2 v/v% in ethanol) for 20 min. It is then washed alternately with ethanol and DI water and dried with nitrogen.
3. The LPG is immersed in glutaraldehyde (50 wt% in H_2_O) for 2 h. Then, it is washed with DI water.
4. The LPG is immersed in a solution of bio-receptors (1 mg/ml in PBS buffer) for 2 h. Anti-κ and anti-λ are used to measure IgG_κ_ and IgG_λ_, respectively. Anti-IgG is used to measure both.

**FIGURE 3:**
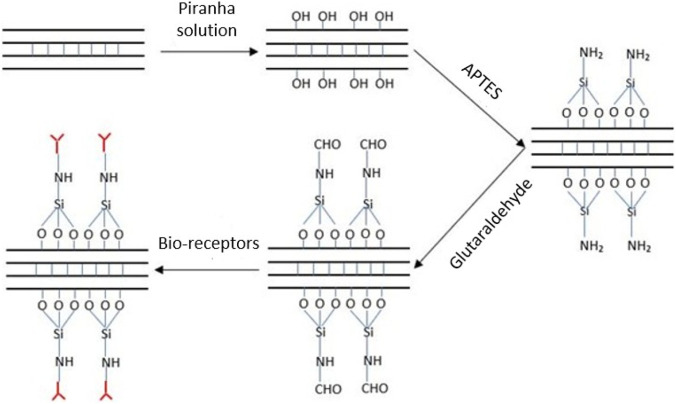
Immobilization of the bio-receptors on the surface of an LPG using glutaraldehyde.

The process is illustrated in Figure 3.

The sensitivities of the IgG_κ_ and IgG_λ_ sensors are analyzed in solutions of the target analyte, with concentrations ranging from 5 μg/mL to 40 μg/mL. After an immersion period of approximately 25 min, the old solution is removed using a suction pump (Flaem, Italy), and a new solution is pipetted into the fiber bath.

The selectivity of the IgG_κ_ and IgG_λ_ sensors is evaluated by comparing the sensors’ responses to the target analytes, the cross-sensitivity to HSA, and the cross-sensitivity to the other interfering M-protein. First, the sensors are immersed in an 80 μg/mL HSA solution. After waiting for 25 min, the HSA solution is suctioned out. Second, the sensor is immersed in 80 μg/mL of the interfering M-protein (IgG_κ_ interferes in the IgG_λ_ sensor, and IgG_λ_ interferes in the IgG_κ_ sensor). After waiting for 25 min, the old solution is suctioned out. Third, the sensor is immersed in a solution with 80 μg/mL of the target analyte. The immersion period is 25 min. Since κ and λ chains exist in all types of antibodies, other antibodies, such as IgM_κ_ and IgM_λ_, can interfere with sensors developed using anti-κ and anti-λ, respectively. Similarly, the IgG_κ_ and IgG_λ_ sensors fabricated with anti-κ and anti-λ can be affected by other types of M-proteins in human serum. For example, IgM_λ_ and IgM_κ_ can affect the measurement of IgG_λ_ and IgG_κ_, respectively. Therefore, additional measurements are required to determine the type of M-protein. Anti-IgG (F_c_ specific) is used to fabricate a whole IgG sensor. The sensor is used to measure both IgG_κ_ and IgG_λ_. The selectivity of a sensor is calculated using Equation 2 (Tonezzer et al., 2019).

$$Selectivity=\frac{R_{t}}{R_{I}}.$$ (2)

Here, $R_{t}$ is the response to the target analyte, and $R_{I}$ is the response to the interfering analyte.

The limit of detection (LoD) is calculated using Equation 3 (Armbruster and Pry, 2008).

$$LoD=\frac{{3.3S}_{y}}{S}.$$ (3)

Here, $S_{y}$ is the standard deviation of the sensor’s signal at baseline, and S is the slope of the calibration curve at baseline.

While the approximately 25-min response time of the current sensor represents a significant improvement over the clinical gold-standard methods such as immunofixation electrophoresis—which typically requires several hours to produce results—there remains substantial opportunity for further optimization. The current detection time is primarily limited by the reliance on static incubation in a macroscopic Teflon fiber bath, which depends on passive diffusion. In future iterations, this response time can be markedly reduced by integrating the LPG sensor with an active microfluidic pumping system. Implementing active microfluidics will significantly enhance the mass transport of target analytes directly to the functionalized sensor surface, thereby accelerating antigen–antibody binding kinetics and advancing the platform toward a truly rapid POCT device.

## 3 Results

For the IgG_κ_ and IgG_λ_ sensors, the complete optical spectra during sensor fabrication and measurement, the real-time wavelength shifts, comparisons between the responses to the target and interfering analytes, and the calibration curves are presented. For the whole IgG sensor, the complete optical spectra during fabrication and measurement, real-time wavelength shifts, and calibration curves for both IgG_κ_ and IgG_λ_ are presented.

### 3.1 IgG_κ_ sensor

**FIGURE 4:**
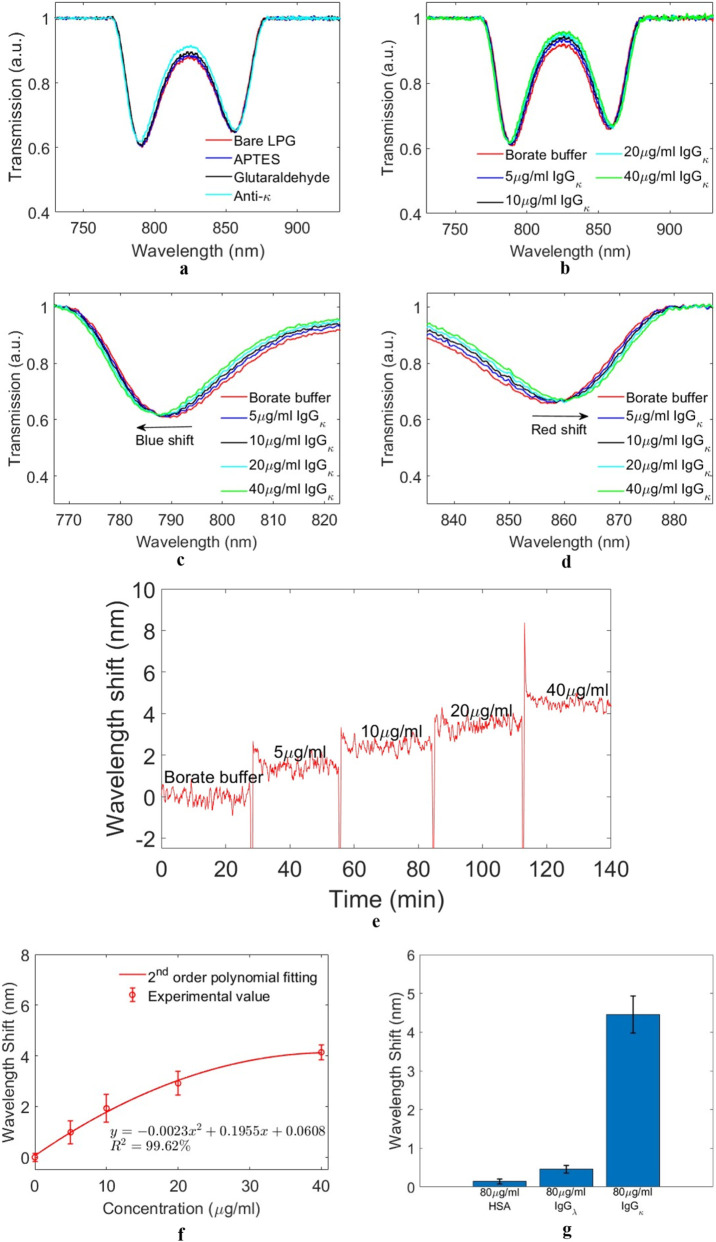
IgG_κ_ sensor. **(a)** Transmission spectra during the surface functionalization of the LPG. **(b)** Transmission spectra during the measurement of IgG_κ_. **(c)** Blue shift of the attenuation band near 790 nm. **(d)** Red shift of the attenuation band near 860 nm. **(e)** Real-time wavelength shift during the measurement. **(f)** Calibration curve of the IgG_κ_ sensor. **(g)** Selectivity test on the IgG_κ_ sensor.

The transmission spectra of a bare LPG and an LPG after surface functionalization are shown in Figure 4a. There are two attenuation bands in the transmission spectrum. As APTES, glutaraldehyde, and anti-κ are deposited on the surface, the attenuation bands move oppositely, and the separation between them increases. The sensor’s transmission spectrum changes with IgG_κ_ concentration (Figure 4b). As the concentration increases from 0 to 40 μg/mL, the attenuation bands near 780 nm move to shorter wavelengths (Figure 4c), and the attenuation band near 860 nm moves to longer wavelengths (Figure 4d). The real-time wavelength shift is shown in Figure 4e. The calibration curve is achieved with second-order polynomial fitting (Figure 4f). The value of R^2^ is 99.62%.

For all sensor configurations in this study, the calibration curves were fitted using second-order polynomial equations rather than linear regression. This non-linear fitting was chosen to accurately reflect the inherent non-linearity of the LPG sensor response over wider concentration ranges. This behavior is attributed to two primary factors: first, the fundamental phase-matching condition (Equation 1) governing the coupling between the core and cladding modes does not produce a perfectly linear wavelength shift with respect to external refractive index changes; second, as the target analyte concentration increases, the available bio-receptor binding sites on the functionalized sensor surface gradually begin to saturate, leading to a characteristic plateauing effect in the optical response at higher concentrations.

A comparison of the sensor’s responses to HSA, IgG_λ_, and IgG_κ_ is shown in Figure 4g. The selectivity is 9.73, which is calculated using Equation 2. The LoD is 2.78 μg/mL, which is calculated using Equation 3.

### 3.2 IgG_λ_ sensor

**FIGURE 5:**
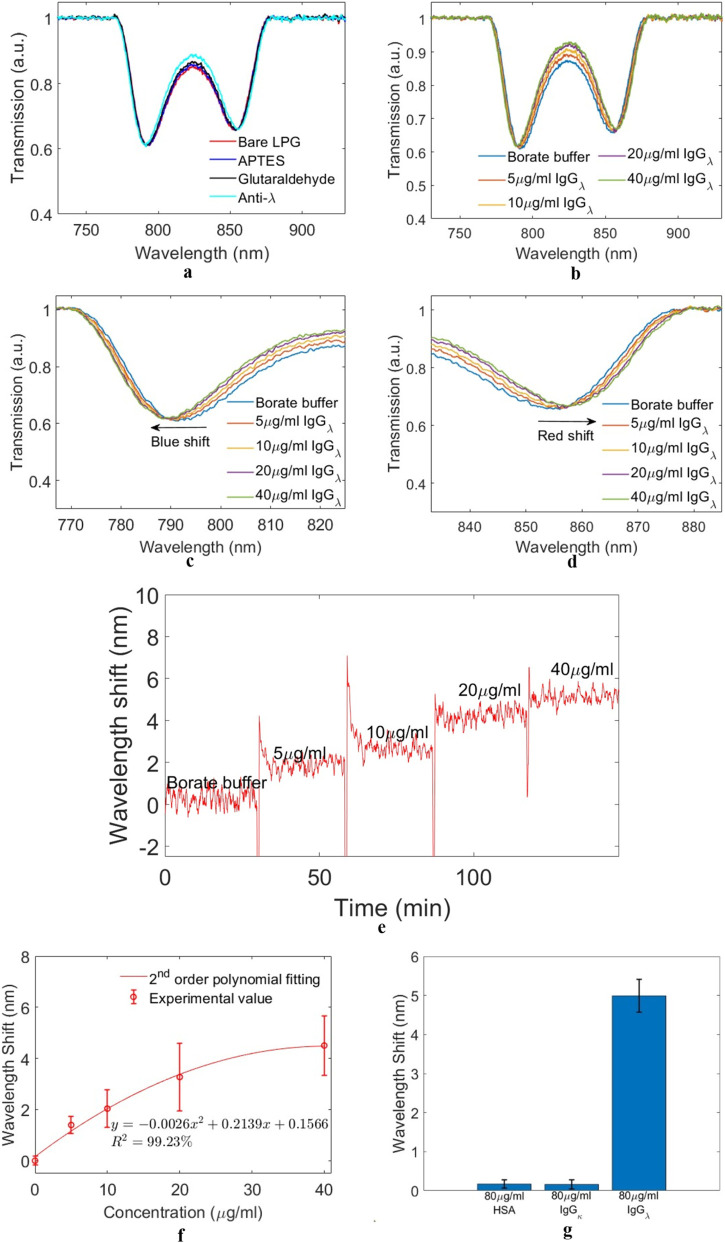
IgG_λ_ sensor. **(a)** Transmission spectra during the surface functionalization of the LPG. **(b)** Transmission spectra during the measurement of IgG_λ_. **(c)** Blue shift of the attenuation band near 790 nm. **(d)** Red shift of the attenuation band near 860 nm. **(e)** Real-time wavelength shift during the measurement. **(f)** Calibration curve of the IgG_λ_ sensor. **(g)** Selectivity test on the IgG_λ_ sensor.

There are two attenuation bands in the transmission spectrum. They shift in opposite directions, and the separation between them increases after surface functionalization (Figure 5a). The transmission spectrum changes with the IgG_λ_ concentration (Figure 5b). The attenuation band near 790 nm shifts to shorter wavelengths (Figure 5c) when the IgG_λ_ concentration increases, and the attenuation band near 860 nm moves to longer wavelengths (Figure 5d). The wavelength shift changes from 0 to nearly 5 nm as the IgG_λ_ concentration changes from 0 to 40 μg/mL (Figure 5e). The calibration is performed using a second-order polynomial fit (Figure 5f), with an R^2^ value of 99.23%. A comparison of the sensor’s response to IgG_λ_ with its responses to HSA and IgG_κ_ is shown in Figure 5g. The selectivity of the sensor is 29.7, as calculated using Equation 2. The LoD of the sensor is 2.75 μg/mL, as calculated using Equation 3.

### 3.3 Measuring IgG_κ_ and IgG_λ_ with the whole IgG sensor

**FIGURE 6:**
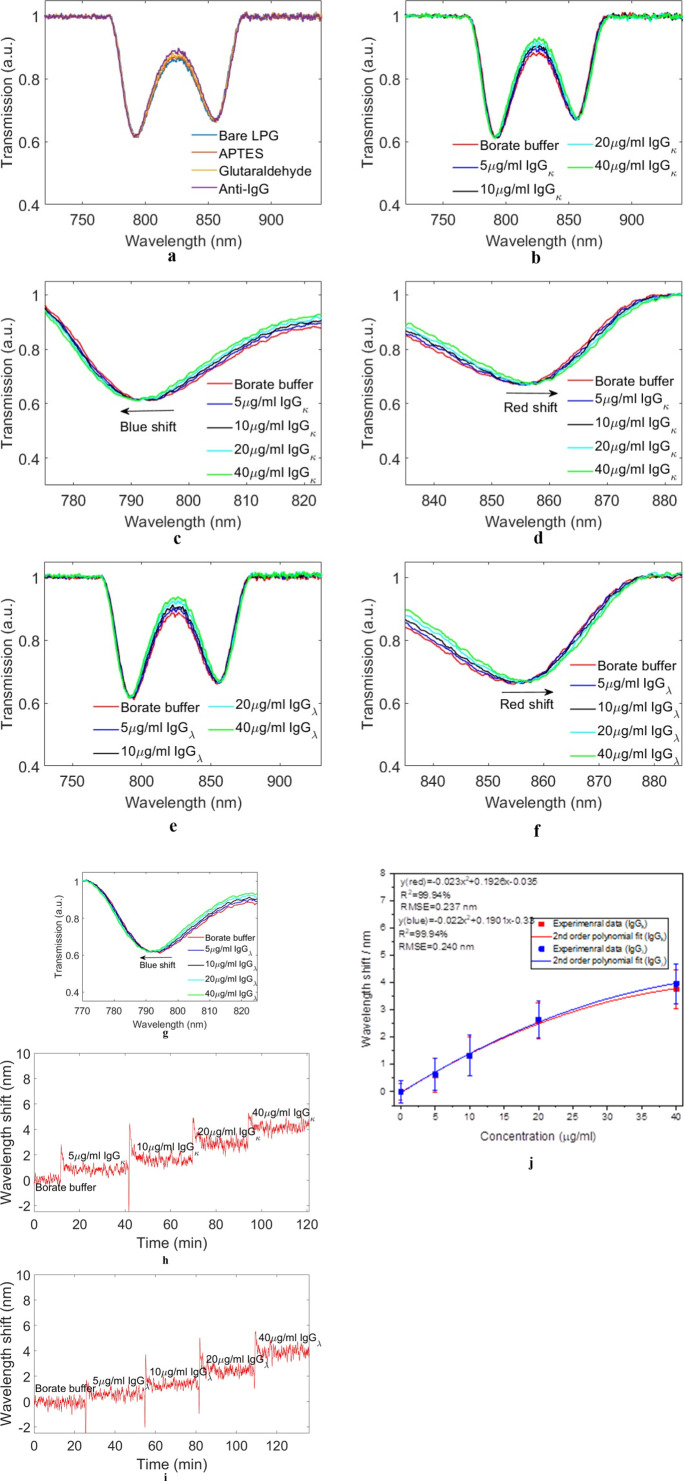
Measurement of IgG_κ_ and IgG_λ_ using a whole IgG sensor. **(a)** Transmission spectra during the surface functionalization of LPG. **(b)** Transmission spectra during the measurement of IgG_κ_. **(c)** Blue shift of the attenuation band near 790 nm during IgG_κ_ measurement. **(d)** Red shift of the attenuation band near 860 nm during IgG_κ_ measurement. **(e)** Transmission spectra during the measurement of IgG_λ_. **(f)** Blue shift of the attenuation band near 790 nm during IgG_λ_ measurement. **(g)** Red shift of the attenuation band near 860 nm during IgG_λ_ measurement. **(h)** Real-time wavelength shift during IgG_κ_ measurement. **(i)** Real-time wavelength shift during IgG_λ_ measurement. **(j)** Calibration curves of the measurements of IgG_κ_ (red) and IgG_λ_ (blue). Error bars shown represent 95% confidence intervals (calculated using the Student’s t-distribution for n = 3 independent measurements).

There are two attenuation bands in the transmission spectrum. As shown in Figure 6a, during surface functionalization, the two attenuation bands shift in opposite directions, and the separation between them increases after each coating layer (APTES, glutaraldehyde, and anti-IgG). The two attenuation bands continue to move farther apart as the concentration of IgG_κ_ (Figure 6b) or IgG_λ_ (Figure 6e) increases. The band near 780 nm moves to shorter wavelengths (Figures 6c,f), and the band near 880 nm (Figures 6d,g) moves to longer wavelengths. The real-time wavelength shifts during the measurements of IgG_κ_ and IgG_λ_ are shown in Figures 6h,f, respectively. The calibration curves are achieved with second-order polynomial fitting. The R^2^ values of IgG_κ_ and IgG_λ_ measurements are 99.94% and 99.93%, respectively. The error bars are calculated from the standard deviation of three independent measurements. The LoDs when measuring IgG_κ_ and IgG_λ_ are 2.77 μg/mL and 2.85 μg/mL, respectively, as calculated using Equation 3.

To ensure the statistical robustness of the calibration, a comprehensive error analysis was performed. Error bars in Figure 6 denote the 95% confidence intervals, which were derived using the Student’s t-distribution for n = 3 independent measurements. The true error of the polynomial regression was assessed via the root-mean-square error (RMSE), yielding highly accurate fits for the IgG_κ_ and IgG_λ_ measurements of 0.237 nm and 0.240 nm, respectively. Furthermore, excellent reproducibility was demonstrated, with the sensors exhibiting an average coefficient of variation (CV) of approximately 20% across all measured concentrations and higher precision (CV < 10%) at clinically elevated concentrations.

## 4 Discussion

Due to the structural similarity among different types of M-proteins, the determination of IgG_κ_ and IgG_λ_ requires a sensor with anti-κ/λ receptors and a sensor with anti-IgG receptors. The sensor fabricated with anti-κ can detect IgG_κ_ with an LoD of 2.78 μg/mL. The selectivity of the sensor is 9.73. It responds to IgG_λ_ because the anti-κ receptor can bind to the λ chain, although the binding strength is approximately 10% of that to the κ chain (product specification). The sensor fabricated with anti-λ can detect IgG_λ_ with an LoD of 2.75 μg/mL. The selectivity of the sensor is 29.7, which is much higher than that of the IgG_κ_ sensor. It is most likely because the anti-λ receptor has higher specificity than the anti-κ receptor. The sensor fabricated with anti-IgG can detect the F_c_ of IgG; therefore, it responds to both IgG_κ_ and IgG_λ_ with similar LoDs (IgG_κ_: 2.77 μg/mL, IgG_λ_: 2.85 μg/mL).

As shown in Table 1, surface plasmon resonance (SPR) sensors and LPGs operating near the dispersion turning point (DTP) have achieved ultra-low LoDs in the sub-ng/ml range for total IgG. While such extreme sensitivities are impressive from a photonic standpoint, they are not strictly advantageous for multiple myeloma diagnostics. In plasma cell dyscrasias, serum M-protein concentrations frequently exceed the normal range and can reach up to 30 mg/mL. Sensors with extreme sub-ng/ml sensitivities are highly susceptible to early saturation and require extreme sample dilution, which can introduce pipetting errors and exacerbate matrix effects. The LPG sensor proposed in this work provides a clinically balanced LoD of 2.75 μg/mL–2.78 μg/mL, offering a dynamic range that satisfies current clinical practice for sample dilution. By eliminating the need for bulky equipment and extensive sample preparation, this LPG optical fiber platform represents a significant step toward robust POCT. Its rapid testing capabilities and ease of use offer strong clinical applicability for the real-time bedside monitoring of disease progression.

Furthermore, a distinct advantage of the LPG platform is its structural tunability. If a specific clinical diagnostic route requires an even broader dynamic range to accommodate exceptionally high M-protein concentrations without early saturation, the inherent sensitivity of the sensor can be systematically engineered. By modifying the physical parameters during fabrication—such as adjusting the grating period (Λ) to couple light to a lower-order cladding mode—the refractive index sensitivity can be intentionally decreased. This engineering flexibility allows the sensor’s dynamic range to be custom-tailored to the specific physiological concentrations of the target biomarker. Most importantly, unlike the highly sensitive sensors reported in the literature that measure only the total IgG, our platform represents the first optical fiber biosensor explicitly engineered to differentiate and quantify the specific IgG_κ_ and IgG_λ_ light chains, thereby satisfying the fundamental requirement for subtyping plasma cell dyscrasias.

It should be noted that for final clinical applications, the developed sensor would need to undergo rigorous testing, including recovery studies and dilution experiments using patient serum samples and samples containing interfering biomolecules such as different serum proteins. A focus of this study is to explore the potential of using an optical fiber long-period grating-based biosensor to discriminate between IgG_κ_ and IgG_λ_ proteins, which is successfully demonstrated. To address potential selectivity issues, we can draw on previously reported results that clearly demonstrated the ability of the proposed biosensors to perform in complex serum media (Korposh et al., 2014; Liu et al., 2018) and have a high degree of selectivity toward specific proteins over the range of other structurally similar immunoglobulins (Wu et al., 2021). It should be noted that biosensors based on antigen–antibody interactions are typically not able to monitor decreases in concentration because of irreversible binding. However, different binding receptors can provide this capability (He et al., 2022).

In a practical clinical setting, this sensor system is envisioned to operate through a streamlined two-tier diagnostic workflow that is conceptually analogous to the reflex testing paradigm currently used in immunofixation but executed far more autonomously at the point of care. Initially, a patient’s serum sample—appropriately diluted to mitigate complex matrix effects—is introduced to the whole IgG (F_c_-specific) sensor. This first step acts as a rapid screening gate to quantify the total IgG levels. If the total IgG concentration exceeds the established healthy physiological baseline, flagging a potential monoclonal gammopathy, the clinical workflow proceeds to the second tier. In this typing phase, the sample is analyzed using the anti-IgG_κ_- and anti-IgG_λ_-functionalized LPG sensors. By accurately measuring the absolute concentrations of IgG_κ_ and IgG_λ_, the platform effectively determines the clonality of the M-protein and establishes the IgG_κ_/IgG_λ_ ratio. This ratio is the critical diagnostic metric for tracking disease progression from monoclonal gammopathy of undetermined significance (MGUS) to smoldering or active multiple myeloma (MM).

This completed proof-of-concept study lays a crucial foundation for several immediate future studies. First, while the selectivity and resilience of similar functionalized LPG platforms have previously been validated by our group in complex media (Wu et al., 2021; Korposh et al., 2014; Liu et al., 2021), the next phase of this specific research will involve rigorous validation using real patient-derived serum samples. This will require executing comprehensive recovery and dilution experiments to benchmark the platform’s clinical accuracy directly against central-laboratory mass spectrometry and IFE data. Second, to transition this technology into a true POCT device, future studies will focus on integrating the LPG sensor into an active microfluidic “lab-on-a-chip” system. Replacing the current macroscopic static Teflon bath with active microfluidic pumping will enhance the mass transport kinetics of the analyte to the sensor surface. This engineering progression is expected to significantly reduce the current 25-min response time, minimize the required sample volumes to the microliter scale, and automate the two-tier diagnostic workflow for rapid bedside clinical application.

## 5 Conclusion

Optical fiber LPG sensors to detect IgG-type M-proteins are developed in this study. The IgG_κ_ sensor is fabricated by immobilizing anti-κ receptors on an LPG to bind to the κ chain in IgG_κ_. The LoD of the sensor is 2.78 μg/mL, and the selectivity is 9.73. The IgG_λ_ sensor is developed using anti-λ receptors, which bind to the λ chain of IgG_λ_. The LoD of the IgG_λ_ sensor is 2.75 μg/mL, and the selectivity is 27.9. A whole IgG sensor is fabricated by immobilizing anti-IgG, which binds to the F_c_ region of IgG. The sensor can detect IgG_κ_ and IgG_λ_ with LoDs of 2.77 μg/mL and 2.85 μg/mL, respectively. The determination of IgG-type M-proteins consists of two steps: i) testing the sample with the whole IgG sensor to determine whether the IgG concentration exceeds the normal range and ii) if the IgG concentration exceeds the normal range, testing the sample with the IgG_κ_ or IgG_λ_ sensor to determine the type of M-protein. The concentration of IgG_κ_ or IgG_λ_ in the serum of patients with IgG-type myeloma can reach 30 mg/mL, which is within the measurement range of the LPG optical fiber sensors.

Overall, this work demonstrates a highly sensitive emerging optical technology that directly addresses the demand for advanced POCT systems, thus offering a rapid, easy-to-use alternative with immense clinical applicability for plasma cell dyscrasia management.

## Data Availability

The original contributions presented in the study are included in the article/supplementary material; further inquiries can be directed to the corresponding author.
